# Pharmacokinetics of a Fixed-Dose Combination Product of Dapagliflozin and Linagliptin and Its Comparison with Co-Administration of Individual Tablets in Healthy Humans

**DOI:** 10.3390/pharmaceutics14030591

**Published:** 2022-03-08

**Authors:** Jin-Woo Park, Jong-Min Kim, Ji Hyeon Noh, Kyoung-Ah Kim, Hyewon Chung, EunJi Kim, Minja Kang, Ji-Young Park

**Affiliations:** 1 Department of Clinical Pharmacology and Toxicology, Korea University College of Medicine, Korea University Anam Hospital, Seoul 02841, Korea; parkzinu@korea.ac.kr (J.-W.P.); jmk157@korea.ac.kr (J.-M.K.); njh2535@korea.ac.kr (J.H.N.); kakim920@kumc.or.kr (K.-A.K.); 2 Department of Neurology, Korea University Medical Center, Seoul 02841, Korea; 3 Division of Clinical Pharmacology, Department of Medicine, Vanderbilt University School of Medicine, Nashville, TN 37232, USA; 4 Department of Clinical Pharmacology and Toxicology, Korea University Guro Hospital, Seoul 08308, Korea; hyewonchung@korea.ac.kr; 5 HK Inno.N, Corporation, Seoul 04551, Korea; eunji.kim24@inno-n.com (E.K.); minja.kang@inno-n.com (M.K.)

**Keywords:** dapagliflozin, linagliptin, fixed-dose combination products, bioequivalence

## Abstract

Dapagliflozin, a selective sodium–glucose co-transporter-2 inhibitor, and linagliptin, a competitive, reversible dipeptidyl peptidase-4 inhibitor, are commonly prescribed antidiabetic medications in general clinics. Since there are several merits to combining them in a fixed-dose combination product, this study investigated the pharmacokinetic equivalence between the individual component (IC) and fixed-combination drug product (FCDP) forms of dapagliflozin and linagliptin. A randomized, open-label, single-dose crossover study was conducted. All participants (*n* = 48) were randomly allocated to group A (period 1: ICs, period 2: FCDP) or group B (period 1: FCDP, period 2: ICs), and each group received either a single dose of IN-C009 (FCDP) or single doses of both dapagliflozin and linagliptin. There was no statistically significant difference found between the pharmacokinetic variables of FCDP and IC. The values of estimated geometric mean ratios and the 90% confidence interval for both maximum concentration and area under the plasma drug concentration–time curve were within the range of 0.8–1.25 for both dapagliflozin and linagliptin. The results of the clinical study demonstrated comparable pharmacokinetic characteristics between IC and FCDP forms of dapagliflozin and linagliptin. The combined use of dapagliflozin and linagliptin was safe and tolerable in both formulations.

## 1. Introduction

Dapagliflozin and linagliptin are commonly used medications for the treatment of type-2 diabetes mellitus (T2DM) [1,2,3]. Dapagliflozin acts by selectively inhibiting sodium–glucose co-transporter-2 (SGLT-2) protein in the kidneys, thereby reducing renal glucose reabsorption and increasing the glucose excretion via urine [3,4]. Dapagliflozin is absorbed rapidly and reaches a maximum concentration (C_max_) within 2 h. It has a half-life (t_1/2_) of 8.1–12.2 h, and approximately 65% of dapagliflozin is metabolized by uridine diphosphate glucuronosyltransferase 1A9 [1,5]. Due to its insulin-independent effects, dapagliflozin is used in combination with several other classes of antidiabetic medications [6,7,8,9].

Linagliptin is a competitive, reversible dipeptidyl peptidase (DPP)-4 inhibitor that increases the levels of active glucagon-like peptide-1 (GLP-1) [10]. GLP-1, an incretin hormone secreted by the small intestine, regulates blood glucose levels by stimulating glucose-dependent postprandial insulin secretion and inhibiting glucagon secretion. Because it is rapidly degraded by DPP-4, the use of DPP-4 inhibitors eventually increases the GLP-1 levels and prevents high blood glucose levels [11,12,13]. The C_max_ of linagliptin is reached within approximately 90 min, and a steady-state level is reached within 4 days at a therapeutic dose (5 mg) [12]. Linagliptin is a known substrate for the cytochrome P450 3A4 enzyme and P-glycoprotein (P-gp) in humans, and its oral bioavailability is approximately 30% [12].

The combined use of dapagliflozin and linagliptin for managing T2DM is reasonable and attractive because of their different but complementary mechanisms of action and separate paths of degradation (i.e., metabolism), thereby avoiding possible drug interactions, which is important for harnessing drug pharmacodynamics and reducing the risk of unexpected adverse events [14,15,16,17]. Compared with a DPP-4 inhibitor, the combined use of SGLT-2 inhibitor and DPP-4 inhibitor is significantly associated with a decrease in glycemic control, body weight, and systolic blood pressure, and their advantages have already been proven for both initial combination and stepwise approaches [14,15]. However, the FCDP for dapagliflozin and linagliptin have not yet been tested. 

Therefore, this study aimed to evaluate the pharmacokinetics and safety of the fixed-combination drug products (FCDPs) of dapagliflozin (10 mg) and linagliptin (5 mg), which were developed to reduce the burden of requiring multiple tablets and thus increasing compliance in healthy participants [18]. 

## 2. Materials and Methods

Sixty-three healthy male volunteers (age, 19–45 years; body weight > 50 kg) agreed to participate in the study and signed a written informed consent form. Only male participants were recruited to avoid the potential risk of pregnancy [19]. The participants were considered healthy after a detailed physical examination by physicians involving 12-lead electrocardiographs (ECG), vital sign assessments, and laboratory evaluations, including blood chemistry, hematology, and urinalysis. The exclusion criteria were a history or evidence of hepatic, renal, gastrointestinal, or hematological abnormalities; hepatitis B, hepatitis C, syphilis, or HIV infection; a history of hypersensitivity to dapagliflozin and/or linagliptin; clinically significant allergic disease; alcohol or drug abuse; heavy smoking (more than 10 cigarettes per day); and use of any medication within 30 days before the start of the study that may have affected the study results. The study protocol was approved (IRB No.2019AN0538, clinicaltrial.gov; NCT05066516) by the Institutional Review Board of Anam Hospital, Korea University Medical Center (Seoul, Korea), and all procedures were conducted following the principles described in the Declaration of Helsinki and Good Clinical Practice guidelines. 

This study was conducted as a randomized, open-label, single-dose crossover study. All participants were randomly allocated to group A (period 1: individual components (ICs), period 2: FCDP) or group B (period 1: FCDP, period 2: ICs). Each group was administered a single dose of IN-C009 (FCDP, dapagliflozin 10 mg/linagliptin 5 mg) (HK Inno.N, Corporation, Seoul, Korea) or co-administered a single dose of dapagliflozin (Forxiga^®^ 10 mg, AstraZeneca, Cambridge, UK) and linagliptin (Trajenta^®^ 5 mg, Beringer-Ingelheim, Ingelheim, Germany) after at least 10 h of overnight fasting. After the 28-day washout period, the participants received the other treatment (group A: IN-C009; group B: dapagliflozin and linagliptin). All medications were biopharmaceutical classification system (BCS) III drugs with high solubility and low permeability. The in vitro dissolution behavior of the formulation was tested in four different pH conditions (pH 1.2, pH 4.0, pH 6.8, and aqueous water) and the results were comparable in both ICs and FCDP (greater than 80% dissolution in 30 min). For FCDP, microcrystalline cellulose and copovidone were used as major excipients. The doses of dapagliflozin and linagliptin used in this study were commercially used and the currently recommended dose for the control of T2DM was used. Previous studies have demonstrated no possible food effect on linagliptin and dapagliflozin; therefore, the food effect was not assessed in this study [20,21].

On day 1 (the day of each drug administration), serial blood samples were drawn immediately before (0 h) and 0.25, 0.5, 0.75, 1, 1.5, 2, 2.5, 3, 4, 6, 8, 12, 24, 48, and 72 h after each dosing to assess the pharmacokinetics of each drug. The collected samples were centrifuged (at 1977× *g*, 4 °C) for 15 min, and the extracted plasma samples were stored frozen at −60 °C until they were analyzed. Plasma dapagliflozin and linagliptin concentrations were determined using high-performance liquid chromatography with tandem mass spectroscopy (LC–MS/MS) described elsewhere, with minor modifications, following the Korea Ministry of Food and Drug Safety and US Food and Drug Administration guidelines [22,23]. 

For dapagliflozin analysis, 100 µL plasma was added to a glass tube containing an internal standard (10 µL of 1 mg/mL dapagliflozin-d_5_) and 1 mL methanol. The samples were vigorously vortexed and centrifuged at 10,770× *g* for 3 min. Next, 100 µL of the upper layer was transferred to a polypropylene tube with 10 µL of internal standard (dapagliflozin-d_5_, Toronto Research, Toronto, ON, Canada) and 300 µL of acetonitrile. After vigorous vortexing and centrifugation at 2191× *g* for 1 min, 200 µL of the upper layer was transferred to another polypropylene tube with 200 µL of ammonium acetate (0.1%, *w*/*v*). A 20 µL aliquot of the solution was finally injected into the LC–MS/MS system; 0.1% ammonium acetate and methanol were used for the mobile phase. The gradient elution mode was applied (methanol proportion ranging between 57.5% and 90% in 5 min) with a constant flow rate of 0.4 mL/min. Dapagliflozin was quantified using the multiple reaction monitoring (MRM) mode. The produced transitions were *m*/*z* 426.47 → 166.84 for dapagliflozin and *m*/*z* 431.50 → 166.85 for dapagliflozin-d_5_. The linear function of dapagliflozin concentration ranged from 1 to 400 ng/mL with regression correlation coefficients of the calibration curves (R) greater than 0.999. The intra-day and inter-day CV values were below 15%. The lower limit of quantification (LLOQ) using this method was 1 ng/mL.

Linagliptin-^13^C-d_3_ (TLC Pharmaceutical Standards Ltd., Newmarket, ON, Canada) was used as the internal standard, and 100 µL of plasma was added to a glass tube containing the internal standard (10 µL of 1 mg/mL linagliptin-^13^C-d_3_) and 1 mL methanol. The samples were vigorously vortexed and centrifuged at 10,770× *g* for 3 min. Next, 100 µL of the upper layer was transferred to a polypropylene tube with 10 µL of the internal standard and 400 µL of methanol. After vigorous vortexing and centrifugation at 2191× *g* for 1 min, 100 µL of the upper layer was transferred to another polypropylene tube containing 300 µL of formic acid in distilled water (0.1%, *w*/*v*). A 20 µL aliquot of the solution was finally injected into the LC–MS/MS system; 0.1% ammonium formate in distilled water and acetonitrile was used as the mobile phase. The gradient elution mode was applied (acetonitrile percentage ranging between 25% and 90% in 4 min) with a constant flow rate of 0.3 mL/min. Linagliptin was quantified using MRM mode. The produced transitions were *m*/*z* 473.18 → 420.20 for linagliptin and *m*/*z* 477.20 → 420.20 for linagliptin-^13^C-d_3_. A linear function of linagliptin concentration ranged from 0.04 to 25 ng/mL with regression correlation coefficients of the calibration curves (R) greater than 0.999. The intra-day and inter-day CV values were <15%. The LLOQ with this method was 0.04 ng/mL.

The pharmacokinetic variables of dapagliflozin and linagliptin were estimated by non-compartmental methods using Phoneix^®^ Winnolin^®^ software (version 8.1, Certara™, Princeton, NJ, USA). The variables included were peak plasma concentration (C_max_), area under the plasma drug concentration–time curve (AUC), time to maximal concentration, terminal elimination half-life (t_1/2_), and oral clearance (CL/F). The AUC from time zero to the last measurable concentration (AUC_last_) was obtained using the trapezoidal rule and AUC from time zero to infinity (AUC_inf_) was calculated as AUC_last_ + C_t_/k_e_ (C_t_, the last plasma concentration measured; k_e_, the elimination rate constant).

SAS statistical software (version 9.4, SAS Institute, Cary, NC, USA) was used for statistical analyses. Mixed-effects models were used to compare pharmacokinetic variables, using treatment, period, and sequence as fixed effects and sequence-nested subjects as random effects. Point estimates of GMRs and two-sided 90% CIs were calculated. The 90% CIs of GMR between 0.8 and 1.25 after comparing the log-transformed data of FCDP and ICs were considered equivalent according to the US Food and Drug Administration guidelines [24]. The safety and tolerability were assessed by vital signs, physical examinations, laboratory tests (hematology, biochemistry, and urinalysis), and 12-lead ECGs were assessed during the study period. Adverse events (AEs) were monitored based on the participants’ self-reporting and general physical examinations. 

## 3. Results

Forty-eight healthy male participants were enrolled in this study. The demographic characteristics of the study participants are summarized in Table 1. The mean age and weight of the participants were 27.3 years and 73.9 kg, respectively. Two participants dropped out due to personal reasons, and a total of 46 participants completed the study. No serious adverse events or clinically significant changes were observed through the safety parameters during the study period. Only one adverse event was reported and resolved spontaneously (myalgia). 

**Figure 1:**
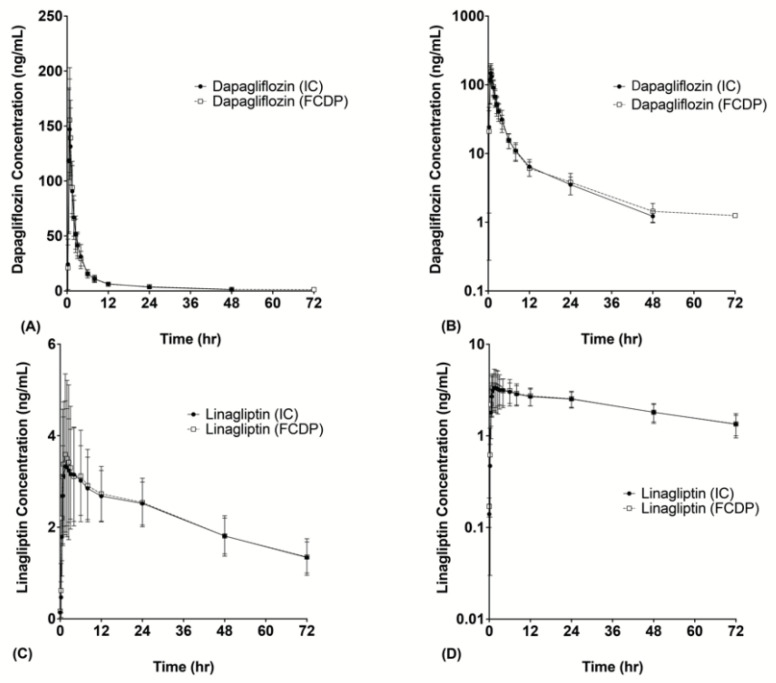
Mean plasma concentration–time profiles of IC (individual component) and FCDP (fixed-combination drug product) forms of dapagliflozin (**A**,**B**) and linagliptin (**C**,**D**). Linear plot, (**A**,**C**); semi-log transformed plot, (**B**,**D**) for *y*-axis.

The mean plasma concentration versus time profile for dapagliflozin and linagliptin (both ICs and FCDP) are presented in Figure 1. 

When we compared the pharmacokinetic variables of dapagliflozin and linagliptin in different formulations, no statistically significant differences were found (Table 2 and Table 3). 

**Figure 2:**
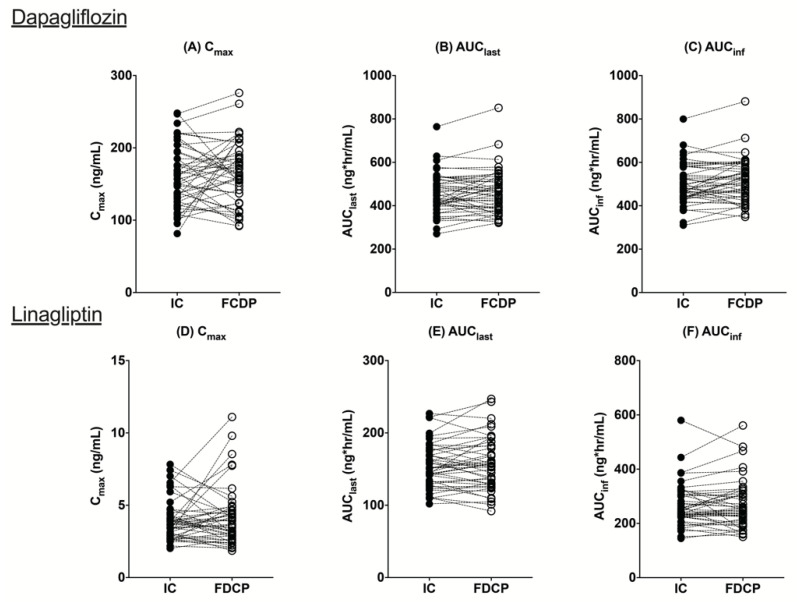
The comparisons of individual C_max_, AUC_last_, and AUC_inf_ of dapagliflozin (**A**–**C**) and linagliptin (**D**–**F**) in IC (individual component) and FCDP (fixed-combination drug product).

The mean C_max_ values for dapagliflozin were 161.32 ng/mL (IC) and 167.59 ng/mL (FCDP) and for linagliptin, 3.95 ng/mL (IC) and 4.2 ng/mL (FCDP). The mean area under the curve (AUC)_last_ values were also comparable for both dapagliflozin (IC: 455.65 ng∙h/mL, FCDP: 465.76 ng∙h/mL) and linagliptin (IC: 155.35 ng∙h/mL, FCDP: 157.37 ng∙h/mL). The estimation values of geometric mean ratios (GMRs) and 90% confidence interval (CI) for both C_max_ and AUCs were within the range of 0.8–1.25 for both dapagliflozin and linagliptin, indicating that FCDP and IC tablets are bioequivalent (Table 4). Individual comparisons of the pharmacokinetic parameters are presented in Figure 2. The intra-participant coefficient of variation (CV) (%) for dapagliflozin was 20.49% for C_max_ and 7.91% for AUC_last_, and 27.58% and 7.96% for linagliptin, respectively. 

## 4. Discussion

The combination treatment using dapagliflozin and linagliptin is reasonable considering their complementary effects due to the different mechanisms of action, different metabolism, and relatively fewer adverse events, including hypoglycemia [14,25,26]. Although dapagliflozin and linagliptin are substrates of P-gp transporter, several clinical studies have already demonstrated that a combination of SGLT-2 inhibitors and DPP-4 inhibitors did not show any significant drug–drug interactions (DDI) [17,27,28]. Assuming that linagliptin and dapagliflozin have similar chemical structures, the possibility of DDI via P-gp may not be significant. Additionally, the Km of P-gp associated linagliptin transport is 187 μM and it does not inhibit P-gp at the therapeutic levels [29]. In vitro studies have shown that dapagliflozin is a weak substrate but not an inhibitor of P-gp [30] and its interaction with other P-gp substrates, including linagliptin, has not yet been reported. 

A recent study suggested that dapagliflozin may have a better outcome in reducing heart failure in T2DM than empagliflozin (4.9 person-years in dapagliflozin vs. 9.0 person-years in empagliflozin) [31]. Both dapagliflozin and empagliflozin have favorable effects on heart failure; however, dapagliflozin is characterized by a longer pharmacological effect and lower SGLT2:SGLT1 receptor selectivity (i.e., higher selectivity of SGLT1 receptors, thereby reducing postprandial blood glucose variations) than empagliflozin [31,32]. Among the DPP-4 inhibitors, linagliptin has a relatively longer half-life and higher potency of DPP-4 inhibition (approximately 90%), whereas saxagliptin has a shorter half-life (parent: 2.5 h, metabolite: 3.1 h) and lower inhibitory effect on DPP-4 (approximately 80%) [27]. Linagliptin also has an advantage over other DPP-4 inhibitors in diabetic patients with renal impairment (kidney excretion: 5%) [33,34,35,36]. Therefore, the development of FCDP for dapagliflozin and linagliptin is likely to have many advantages in the clinical management of uncontrolled T2DM. 

The results of this study showed that the pharmacokinetic profiles after administration of individual dapagliflozin and linagliptin tablets were comparable to the FCDP form. In other words, the systemic exposure to IC and FCDP forms of dapagliflozin and linagliptin were similar in terms of C_max_ and AUCs. Both treatments were well-tolerated without significant adverse events and showed acceptable intra-subject variability [37]. The observed C_max_ values for dapagliflozin were 161.32 ng/mL (IC) and 167.59 ng/mL (FCDP), which were reached at 0.75 h for both, suggesting similar absorption profiles for dapagliflozin in the two formulations. The extent of absorption (i.e., AUC) too was very similar for both formulations (IC: 455.65 ng∙h/mL, FCDP: 465.76 ng∙h/mL). Similar results were also obtained for linagliptin (C_max_: 3.95 ng/mL in IC and 4.2 ng/mL in FCDP, AUC_last_: 155.35 ng∙h/mL in IC and 157.37 ng∙h/mL in FCDP). These results indicate the successful manufacture of the FCDP form of dapagliflozin and linagliptin combined [38]. 

FCDP is defined as a combination of more than two active chemicals in a single pharmaceutical administration [24,38]. The advantage of FCDP is mainly cost-effectiveness and increasing compliance by reducing the number of total medications administered at once [39]. Inappropriately manufactured FCDPs can result in reduced effectiveness or enhanced toxicity in routine clinical practice [40]. Although this study did not contain pharmacodynamic data, including blood glucose levels or HbA1c, it is believed that this FCDP should be generally comparable to its IC forms, considering that pharmacodynamic results generally correlate with pharmacokinetic variables and there were no significant adverse events related to the medication [41]. Indeed, the GMRs of the log-transformed ratio for dapagliflozin were 1.0413 (0.9554–1.1349) for C_max_ and 1.0219 (0.9885–1.0564) for AUC_last_; those for linagliptin were 1.0265 (0.9141–1.1526) and 1.0062 (0.9731–1.0404), respectively, which were within the predefined bioequivalence range. This showed that ICs and FCDP exhibited comparable pharmacokinetic characteristics [24]. 

## 5. Conclusions

The results of this clinical study demonstrated similar pharmacokinetic characteristics between IC and FCDP forms of dapagliflozin and linagliptin at commercially used dosages of 10 mg and 5 mg, respectively. These results met the pharmacokinetic bioequivalence criteria. The combination of dapagliflozin and linagliptin was safe and tolerable in both formulations. 

## Figures and Tables

**Table 1:** Baseline demographic characteristics.

Parameters	Mean ± SD	Min	Max	Median
Age (years)	27.3 ± 5.8	19	45	26
Weight (kg)	73.9 ± 10.2	53.5	99.7	72.6
Height (cm)	174.7 ± 5.2	161	185	175
BMI (kg/m^2^)	24.2 ± 2.9	18.3	29.8	25.1

**Table 2:** Pharmacokinetic parameters of dapagliflozin in different formulations.

	IC	FCDP
t_1/2_ (h)	11.34 ± 3.39	12.76 ± 3.84
C_max_ (ng/mL)	161.32 ± 42.05	167.59 ± 42.09
t_max_ (h)	0.75 (0.5–4)	0.75 (0.5–2.5)
AUC_last_ (ng·h/mL)	455.65 ± 92.84	465.76 ± 99.29
AUC_inf_ (ng·h/mL)	500.34 ± 93.93	516.46 ± 98.28
CL/F (L/h)	20.67 ± 3.89	20.01 ± 3.6

Notes: All values are expressed as the mean ± SD, except for t_max_, which is shown as the median (range). Abbreviations: IC, individual component; FCDP, fixed-combination drug product; t_1/2_, half-life; C_max_, peak plasma concentration; t_max_, time to C_max_; AUC_last_, area under the plasma concentration–time curve from 0 to the time of the last measurable concentration (72 h); AUC_inf_, area under the plasma concentration–time curve from 0 to infinity; t_1/2_, elimination half-life; CL/F, oral clearance.

**Table 3:** Pharmacokinetic variables of linagliptin in different formulations.

	IC	FCDP
t_1/2_ (h)	54.94 ± 13.6	54.29 ± 12.26
C_max_ (ng/mL)	3.95 ± 1.42	4.2 ± 2.01
t_max_ (h)	2.5 (0.75–24)	1.5 (0.5–12)
AUC_last_ (ng·h/mL)	155.35 ± 30.35	157.37 ± 35.9
AUC_inf_ (ng·h/mL)	265.55 ± 78.74	267.97 ± 88.03
CL/F (L/h)	40.44 ± 10.50	40.81 ± 11.74

Notes: All values are expressed as the mean ± SD, except for t_max_, which is shown as the median (range). Abbreviations: IC, individual component; FCDP, fixed-combination drug product; t_1/2_, half-life; C_max_, peak plasma concentration; t_max_, time to C_max_; AUC_last_, area under the plasma concentration–time curve from 0 to the time of the last measurable concentration (72 h); AUC_inf_, area under the plasma concentration–time curve from 0 to infinity; t_1/2_, elimination half-life; CL/F, oral clearance.

**Table 4:** Point estimates and 90% CIs for log-transformed pharmacokinetic parameters (C_max_, AUC_last_, AUC_inf_) of dapagliflozin and linagliptin IC vs. FCDP tablets.

Drugs	Variable	GMR (90% CI)	Intra-Participant CV%
Dapagliflozin	C_max_	1.0413 (0.9554–1.1349)	20.49
	AUC_last_	1.0219 (0.9885–1.0564)	7.91
	AUC_inf_	1.0324 (1.0010–1.0648)	7.35
Linagliptin	C_max_	1.0265 (0.9141–1.1526)	27.58
	AUC_last_	1.0062 (0.9731–1.0404)	7.96
	AUC_inf_	0.9996 (0.9477–1.0542)	12.67

Abbreviations: IC, individual component; FCDP, fixed-combination drug product; GMR, geometric mean ratio; CI, confidence interval; C_max_, peak plasma concentration; AUC_last_, area under the plasma concentration–time curve from 0 to 72 h; AUC_inf_, area under the plasma concentration–time curve from 0 to infinity; CV, coefficient of variation.

## Data Availability

The data presented in this study are available upon reasonable request from the corresponding author. The data are not publicly available because of privacy concerns.
